# Flow-controlled ventilation (FCV) improves regional ventilation in obese patients – a randomized controlled crossover trial

**DOI:** 10.1186/s12871-020-0944-y

**Published:** 2020-01-28

**Authors:** Jonas Weber, Leonie Straka, Silke Borgmann, Johannes Schmidt, Steffen Wirth, Stefan Schumann

**Affiliations:** Department of Anesthesiology and Critical Care, Medical Center – University of Freiburg, Faculty of Medicine, University of Freiburg, Freiburg, Germany

**Keywords:** Mechanical ventilation, Obesity, Flow-controlled ventilation, Ventilation modes: pressure waveform

## Abstract

**Background:**

In obese patients, high closing capacity and low functional residual capacity increase the risk for expiratory alveolar collapse. Constant expiratory flow, as provided by the new flow-controlled ventilation (FCV) mode, was shown to improve lung recruitment. We hypothesized that lung aeration and respiratory mechanics improve in obese patients during FCV.

**Methods:**

We compared FCV and volume-controlled (VCV) ventilation in 23 obese patients in a randomized crossover setting. Starting with baseline measurements, ventilation settings were kept identical except for the ventilation mode related differences (VCV: inspiration to expiration ratio 1:2 with passive expiration, FCV: inspiration to expiration ratio 1:1 with active, linearized expiration). Primary endpoint of the study was the change of end-expiratory lung volume compared to baseline ventilation. Secondary endpoints were the change of mean lung volume, respiratory mechanics and hemodynamic variables.

**Results:**

The loss of end-expiratory lung volume and mean lung volume compared to baseline was lower during FCV compared to VCV (end-expiratory lung volume: FCV, − 126 ± 207 ml; VCV, − 316 ± 254 ml; *p* < 0.001, mean lung volume: FCV, − 108.2 ± 198.6 ml; VCV, − 315.8 ± 252.1 ml; *p* < 0.001) and at comparable plateau pressure (baseline, 19.6 ± 3.7; VCV, 20.2 ± 3.4; FCV, 20.2 ± 3.8 cmH_2_O; *p* = 0.441), mean tracheal pressure was higher (baseline, 13.1 ± 1.1; VCV, 12.9 ± 1.2; FCV, 14.8 ± 2.2 cmH_2_O; *p* < 0.001). All other respiratory and hemodynamic variables were comparable between the ventilation modes.

**Conclusions:**

This study demonstrates that, compared to VCV, FCV improves regional ventilation distribution of the lung at comparable PEEP, tidal volume, P_Plat_ and ventilation frequency. The increase in end-expiratory lung volume during FCV was probably caused by the increased mean tracheal pressure which can be attributed to the linearized expiratory pressure decline.

**Trial registration:**

German Clinical Trials Register: DRKS00014925. Registered 12 July 2018.

## Background

In obese patients, the excessive adipose tissue around the thorax and the visceral organs reduce the functional residual capacity and expiratory reserve volume [1]. Obesity also leads to a low respiratory system compliance, early expiratory alveolar collapse with consecutive atelectasis, increased airway resistance [2] and increased risk for airway closure [3]. All these changes make mechanical ventilation in obese patients prone to respiratory complications [4, 5].

An emerging ventilation technique to linearize expiratory flow is flow-controlled ventilation (FCV), provided by the new ventilator Evone (Ventinova Medical B.V., Eindhoven, the Netherlands). This device provides a constant positive flow during inspiration and a constant negative flow during expiration. Thereby pressure increases linearly during inspiration [comparable to volume-controlled ventilation (VCV)] and decreases linearly during expiration. Recently, we demonstrated that linearizing the expiratory flow improved lung recruitment, the homogeneity of lung aeration [6, 7], gas exchange [8] and further attenuated experimental lung injury [9]. Since FCV is a new emerging technique comparative clinical studies in humans, particularly in patients with impaired respiratory system mechanics, are lacking.

We hypothesized that FCV improves regional ventilation distribution of the lung and respiratory system mechanics in obese patients. Therefore, we compared regional ventilation using electrical impedance tomography (EIT) and respiratory system mechanics during FCV and VCV in obese patients in a randomized controlled crossover trial.

## Methods

### Ethics, consent and permission

The study was approved by the Ethics Committee of the University Medical Center of Freiburg (Engelbergstr. 21, 79106 Freiburg, Germany, Ethical Committee N° 179/18) on 29th March 2018 (Chairperson Prof. Dr. R. Korinthenberg) and registered at the German Register for Clinical Trials (DRKS00014925). Please note that this study adheres to the CONSORT guidelines.

### Study design and patient population

In order to cope with potential interindividual variability, the study was designed as a randomized controlled interventional crossover trial. After obtaining written informed consent, we studied twenty-three obese patients with body mass index (BMI) ≥ 30 kg∙m^− 2^. Patients eligible for enrolment were patients with physical status ASA ≤ III undergoing elective bariatric surgery. Exclusion criteria were ASA physical status >III, age < 18 years, pregnancy, emergency procedure, cardiac pacemaker and other active implants, chronic obstructive pulmonary disease classified as GOLD stage > II or refusal to participate. The trial was conducted at the University Medical Center Freiburg, Germany. Participants were enrolled and assigned by a study related anesthesiologist. Data were collected at the University Medical Center of Freiburg, Germany.

### Procedure

After obtaining written informed consent, 23 patients were included in the study. After primary recruitment and preoperative evaluation, the patients received routine monitoring (electrocardiography, SpO_2_, noninvasive blood pressure measurement; Infinity Delta XL, Dräger Medical, Lübeck, Germany) and a 18–20-G intravenous catheter was established. After preoxygenation to an fraction of expired oxygen of 0.8, anesthesia was induced with 0.3–0.5 μg∙kg^− 1^ predicted body weight [10] iv sufentanil (Janssen-Cilag, Neuss, Germany) and 2–3 mg∙kg^− 1^ actual body weight iv propofol (Fresenius Kabi, Bad Homburg vor der Höhe, Germany). Tracheal intubation was facilitated with 0.6 mg∙kg^− 1^ predicted body weight iv rocuronium (Fresenius Kabi). If the patient required a rapid sequence induction, neuromuscular blockage was performed by the administration of 1.0 mg∙kg^− 1^ predicted body weight iv rocuronium. Neuromuscular blockage was monitored with a mechanomyograph (TOFscan; Dräger Medical). For tracheal intubation, we used tracheal tubes with low pressure cuffs (internal diameter of 7.0–7.5 mm for women and 8.0 mm for men; Mallinckrodt Hallo-Contour; Covidien, Neustadt an der Donau, Germany). After adequate placement of the tracheal tube, iv propofol was administered continuously (110–150 μg∙kg^− 1^∙min^− 1^). Potential hypotension (defined as mean arterial pressure < 65 mmHg) was treated with a continuous infusion of iv noradrenaline (0.03–0.2 μg∙kg^− 1^∙min^− 1^). Perioperative volume requirements were addressed with a crystalloid solution (8 ml∙kg^− 1^∙h^− 1^, Jonosteril; Fresenius Kabi). According to our local standard, mechanical ventilation was started as volume-controlled baseline ventilation (Fabius Tiro, Dräger Medical) with a tidal volume of 7 ml∙kg^− 1^ predicted body weight, inspiration-to-expiration ratio of 1:2, a positive end-expiratory-pressure (PEEP) of 9 cmH_2_O and ventilation frequency set to maintain an end-tidal carbon dioxide partial pressure between 4.7 and 5.1 kPa. These ventilation settings were based on our study protocol and in accordance with our clinical routine in obese patients. After 7 min of baseline ventilation, all patients were randomly allocated to one of two crossover groups to receive ventilation sequences either VCV-FCV or FCV-VCV for 7 min per ventilation mode. To avoid irritations due to the surgical procedure (e.g. impaired respiratory mechanics by the capnoperitoneum and electrical irritations of the measurement of Electrical Impedance Tomography), our study was performed prior to the surgical intervention. For adequate allocation, a computer generated randomization in blocks was used. Disclosure of the randomization was requested right after induction of anesthesia. A study related anesthesiologist conducted the randomization in blocks, enrolled participants and assigned participants to the interventions. During the study protocol, ventilation variables were kept constant as set during the baseline measurements. To prevent from the risks of extubation and reintubation, FCV was performed by introducing the narrow-bore tracheal tube (Tribute, Ventinova Medical B.V.) into the standard tracheal tube. Blocking the cuff of the Tritube in the lumen of the tracheal tube provided a sufficient seal. By controlling both tube’s markings, placement of the Tritube’s tip exceeding that of the standard tracheal tube by 2–5 mm was ensured, and the potential risk of bronchial intubation was avoided. Respiratory data were collected from both ventilators via the respective serial communication interface and analyzed offline. Electrical impedance tomography (EIT) was performed with PulmoVista 500 (Dräger Medical) in all patients to measure regional ventilation, changes in relative thoracic electrical impedance during the different ventilation phases, relative end-expiratory lung volume (ΔEELV) and to compare the expiratory decrease in intrapulmonary air [11–13].

### Ventilation modes

**Fig. 1:**
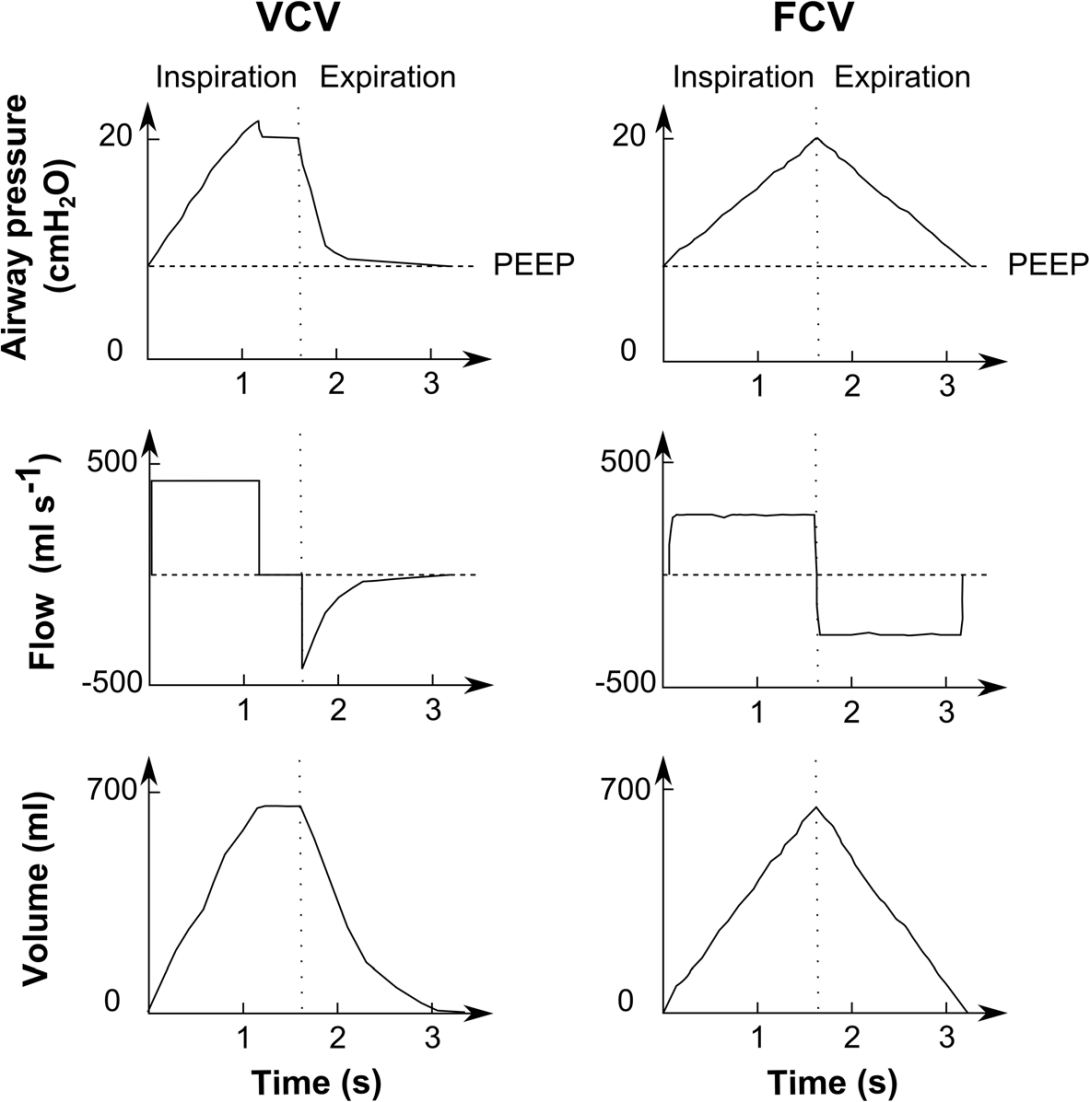
Comparison of flow-controlled ventilation (FCV) with conventional volume-controlled ventilation (VCV)

Ventilation settings during baseline measurements and VCV were identical. In each patient, baseline measurements were performed prior to the intervention. During FCV, patients were ventilated with a constant positive flow during inspiration and a constant negative flow during expiration (Fig. 1). To avoid intrinsic PEEP, the intratracheal pressure is monitored continuously via a dedicated pressure measurement lumen of the Tritube. During FCV, the operator is able to adjust the inspiratory flow rate, inspiration to expiration ratio, peak inspiratory pressure, end-expiratory pressure and the inspiratory concentration of oxygen. In this special ventilation mode, there is no direct way to control minute volume via tidal volumes and/or respiratory rate. However, the respiratory rate depends on the peak inspiratory pressure, the set (positive) end-expiratory pressure, the set inspiratory flow rate, the inspiration to expiration ratio and the patient’s lung compliance [14]. The (end) expiratory pressure was kept constant in all conditions during the study procedure.

### End points and data collection

ΔEELV was the primary endpoint of this study. EIT recordings were analyzed using software developed in Matlab (R2014, The MathWorks Inc.). We derived ΔEELV from adjusting end-expiratory impedance changes by tidal volume and tidal impedance changes as described before [7, 11]. As a first step, the lung area estimation method was applied to all EIT recordings to estimate the relevant lung area [15]. Afterwards, global tidal impedance curves were calculated. These curves represent the sum of impedance of all pixels per frame over time. To scale the absolute impedance values to milliliters, the relation between tidal impedance change and tidal volume was used. Changes of the baseline of these tidal impedance curves were determined as estimates for changes of the end-expiratory lung volume. ΔEELV was then calculated as the difference of end-expiratory lung volume during the different ventilation phases [11]. Secondary endpoints were the respiratory system variables: plateau pressure (P_Plat_), mean tracheal pressure (P_mean_), mean tracheal pressure during expiration (P_mean expiration_), peripheral oxygen saturation (SpO_2_), fraction of inspired oxygen (FiO_2_) and quasi-static respiratory system compliance (C_RS_). To calculate C_RS_ during FCV, the plateau pressure was determined from a short (approximately 0.1 s) end-inspiratory pause. This pause is performed automatically by the Evone ventilator (Ventinova Medical B.V.) with every ten breaths and used to calculate C_RS_. Non-invasively collected hemodynamic variables included mean systolic blood pressure, mean diastolic blood pressure, mean arterial pressure and heart rate. To compare relative intrapulmonary air distribution, baseline tidal impedance curves for ventral and dorsal lung areas were determined and compared as described before [7, 12]. The differences in mean lung volume (ΔMLV) between baseline ventilation and VCV and FCV were calculated, respectively. Further, the decrease in global thoracic electrical impedance during each ventilation mode was separated into four equal sections (ΔEI_25_, ΔEI_50_, ΔEI_75_ and ΔEI_100_), then matched with the correlating decrease in tidal volume and compared successively.

Pressure data from the Evone are based on direct tracheal pressure measurement via a dedicated lumen of the Tritube. To allow for comparability of pressure data from both ventilators and to calculate quasi-static compliance of the respiratory system, airway pressure data from the Dräger Fabius Tiro were generally converted into tracheal pressure data by calculating the flow dependent pressure drop across the respective tracheal tube and pointwise subtracting this value from airway pressure [16]. Thus all pressure data in the following refer to the respective tracheal pressure.

The datasets used and analyzed during the current study are available from the corresponding author on request. Please note that EIT data files require large memory.

### Sample size calculation and statistical analysis

In regard to previous investigations on gas exchange during FCV in a porcine model of ARDS [17] and the crossover design (paired test conditions) we assumed a standardized effect size of the primary endpoint of 0.7 (being the quotient of differences in means and SD). To reach a test power of 0.8 and a desired level of significance of 0.05, 19 patients were required. To compensate for potential incomplete data sets, 23 patients were included in the study. Lilliefors tests were used to confirm that the assumed normal distribution cannot be rejected.

Values are presented as mean ± standard deviation, unless indicated otherwise. Statistical analysis was done using Matlab (R2014, The MathWorks Inc., Natick, MA, USA). Linear mixed effects model analyses were performed to check for differences between respiratory variables and variables resulting from EIT measurements during the ventilation phases using R based software (jamovi project (2018), jamovi (Version 0.9.2.3), retrieved from https://www.jamovi.org). For each measured primary and secondary endpoint (dependent variable), the influence of the ventilation mode (baseline ventilation, VCV and FCV) and the ventilation sequence (baseline-VCV-FCV, baseline-FCV-VCV) (factors) was investigated. *P* < 0.05 was considered statistically significant.

## Results

**Fig. 2:**
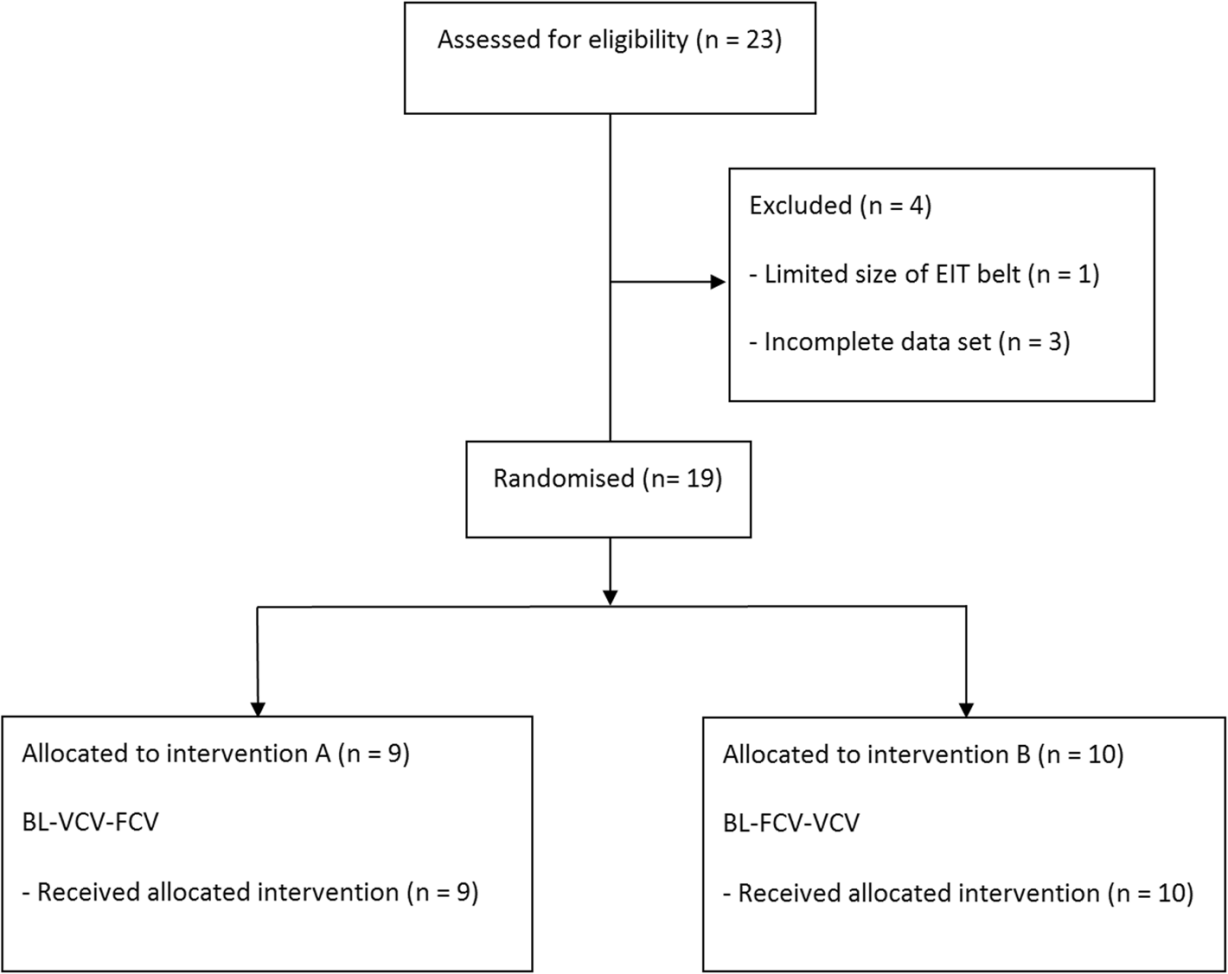
Flow diagram of the study population

In total, 23 consecutive patients presenting for elective bariatric surgery were included and 19 complete data sets could be recorded. Patients were recruited from 30th July 2018 to 23rd October 2018. One patient had to be excluded due to limited size of the EIT belt, three other patients due to incomplete data collection (Fig. 2). There were no adverse events during the study procedure. The study was ended regularly after the last subject was included. Age, gender, ASA physical status, predicted and actual body weight and BMI were comparable between the two interventional groups (Table 1).

**Table 1:** Patients characteristics (*n* = 19)

	Baseline_VCV_FCV (*n* = 9)	Baseline_FCV_VCV (*n* = 10)
Age (yr)	48.6 ± 6.9	43.4 ± 13.3
Gender (n), female/male	6/3	8/2
ASA I/II/III (n)	0/0/9	0/0/10
PBW (kg)	64.2 ± 12.6	59.0 ± 7.0
ABW (kg)	127.0 ± 27.6	120.0 ± 27.1
BMI (kg∙m^−2^)	42.7 ± 4.6	42.5 ± 7.7

Baseline_VCV_FCV, randomization that was characterized by baseline measurements, followed by volume-controlled ventilation (VCV), followed by flow-controlled ventilation (FCV); baseline_FCV_VCV, randomization that was characterized by baseline measurements, followed by flow-controlled ventilation (FCV), followed by volume-controlled ventilation (VCV);* ASA* American Society of Anesthesiologists, *PBW* Predicted body weight, *ABW* Actual body weight, *BMI* Body mass index

**Fig. 3:**
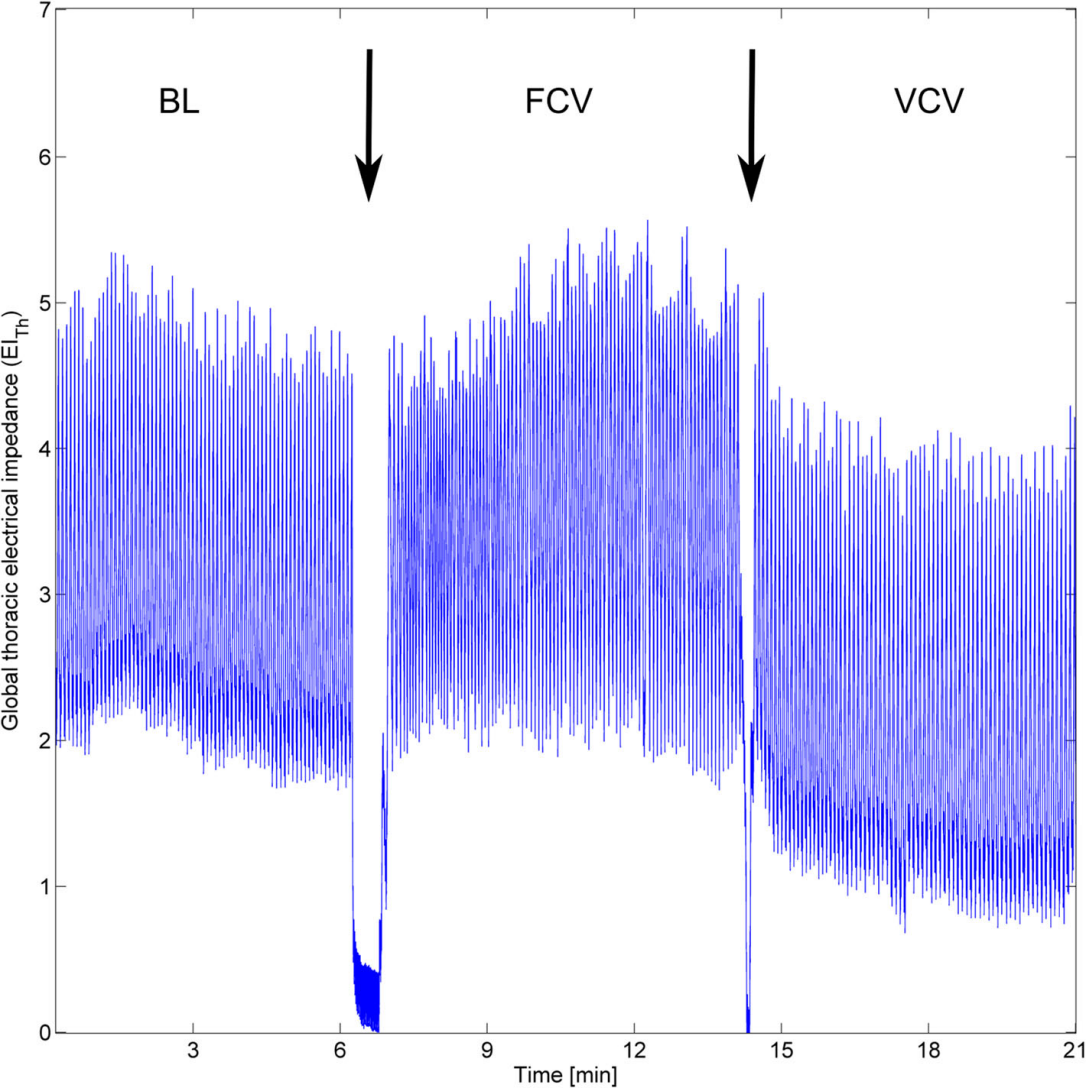
Exemplary relative global thoracic electrical impedance (EI_Th_) of one patient during the study protocol. BL, baseline (volume-controlled) ventilation; VCV, volume-controlled ventilation; FCV, flow-controlled ventilation. The first slope represents the insertion of the Tritube® into the standard tracheal tube. The second slope represents the remove of the Tritube and re-connecting to the Dräger Fabius Tiro ventilator. Arrows indicate the switch between the respective ventilation modes

**Fig. 4:**
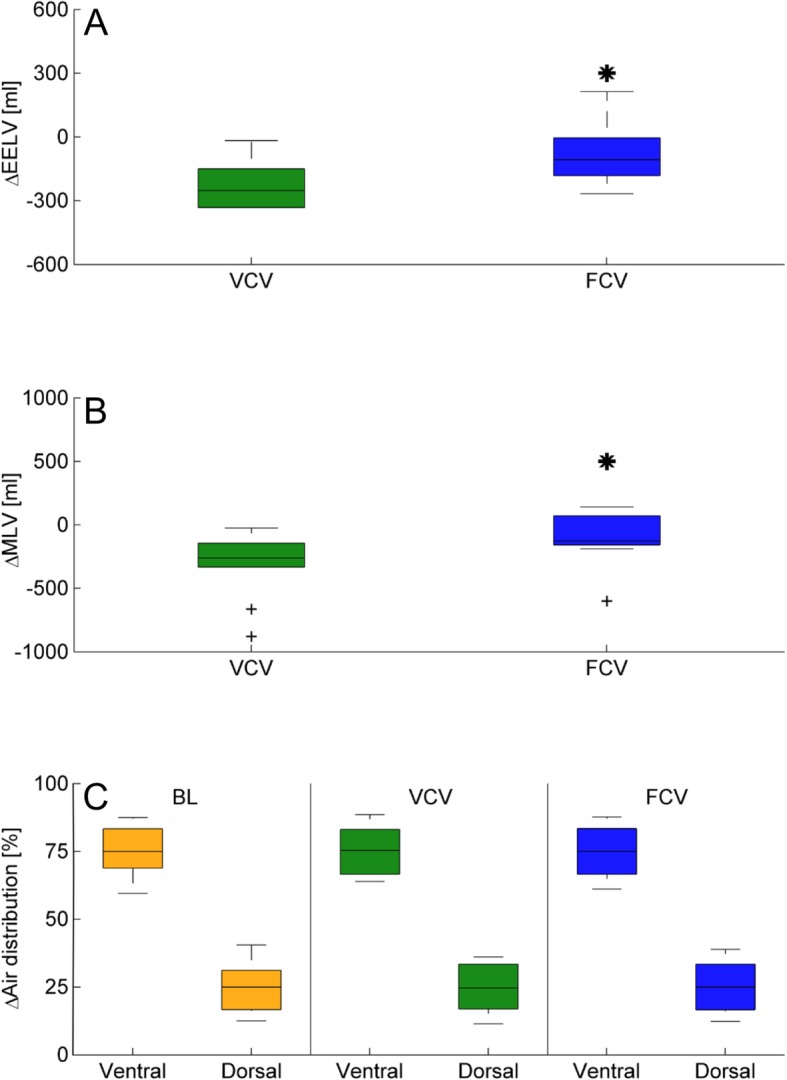
Alteration of end-expiratory lung volume ΔEELV (**a**), mean lung volume ΔMLV (**b**) and comparison in percentage air distribution between ventral and dorsal lung areas (**c**). BL = volume-controlled baseline ventilation, VCV = volume-controlled ventilation and FCV = flow-controlled ventilation. On each box, the central mark indicates the second quartile, the bottom and top edges indicate quartiles (25th percentile and 75th percentile). * = *p* ≤ 0.001 for FCV vs. VCV [linear mixed effect model analyses were used to check for differences between the ventilation phases using R based software (jamovi project 2018, version 0.9.2.3)]. The randomization had no significant effect on the measured difference in end-expiratory lung volume between the ventilation phases

During mechanical ventilation, end-expiratory lung volume decreased generally (Fig. 3). ΔEELV between baseline ventilation and FCV (− 126 ± 207 ml) was lower than between baseline and VCV (− 316 ± 254 ml, *p* < 0.001). ΔMLV between baseline and FCV (− 108 ± 198 ml) was lower than between baseline and VCV (− 315 ± 252 ml, *p* < 0.001) (Fig. 4). P_mean_ and P_mean expiration_ was higher during FCV. No significant differences in tidal volume, ventilation frequency, P_Plat_, SpO_2_ and C_RS_ were found between FCV and VCV. All hemodynamic variables were comparable during FCV and VCV (Table 2).

**Table 2:** Respiratory and hemodynamic variables

Variable	Baseline	VCV	FCV	p_vent_
V_T_ (mL)	440.2 ± 33.0	442.0 ± 33.2	457.4 ± 50.7	0.148
V_T_ PBW (ml∙kg^− 1^)	7.3 ± 1.0	7.3 ± 1.0	7.4 ± 0.8	0.621
VF (min^− 1^)	14.2 ± 2.4	14.1 ± 2.6	14.0 ± 2.5	0.834
P_Plat_ (cmH_2_O)	19.6 ± 3.7	20.2 ± 3.4	20.2 ± 3.8	0.441
P_mean_ (cmH_2_O)	12.9 ± 1.2	13.1 ± 1.1	14.8 ± 2.2*^#^	< 0.001
P_mean expiration_ (cmH_2_O)	10.6 ± 0.1	10.7 ± 0.1	14.2 ± 1.9*^#^	< 0.001
C_RS_ (ml∙cmH_2_O^− 1^)	46.6 ± 17.0	47.2 ± 16.6	44.6 ± 16.3	0.272
Heart rate (min^− 1^)	60.0 ± 11.8	57.5 ± 10.4	57.7 ± 10.0	0.261
Systolic blood pressure (mmHg)	135.0 ± 15.6	131.0 ± 19.2	131.0 ± 12.6	0.343
Diastolic blood pressure (mmHg)	75.8 ± 12.1	74.4 ± 10.4	71.0 ± 11.4	0.132
MAP (mmHg)	95.5 ± 12.0	93.4 ± 12.8	91.0 ± 10.2	0.167
SpO_2_ (%)	99.4 ± 0.96	99.2 ± 1.5	99.1 ± 1.79	0.642
SpO_2_/ FiO_2_ (%)	1.7 ± 0.02	1.7 ± 0.02	1.7 ± 0.03	0.642

Values are stated as mean ± SD. Baseline, baseline measurements (consisting of volume-controlled ventilation); *VCV* Volume-controlled ventilation, *FCV* Flow-controlled ventilation, *V*_*T*_ tidal volume, *V*_*T*_ *PBW* tidal volume per predicted body weight, *VF* Ventilation frequency, *P*_*Plat*_ Plateau pressure, *P*_*mean*_ mean tracheal pressure, *P*_*mean expiration*_ mean tracheal pressure during expiration, *C*_*RS*_ Respiratory system compliance, *MAP* Mean arterial pressure, *SpO*_*2*_ Peripheral oxygen saturation, *FiO*_*2*_ Fraction of inspired oxygen, *p*_*vent*_ p for ventilation mode* = *p* < 0.001 for FCV vs. baseline and for ^#^ = *p* < 0.001 for FCV vs. VCV. Linear mixed effect model analyses were used to check for differences between the ventilation phases using R based software (jamovi project 2018, version 0.9.2.3). The randomization had no significant effect on the measured respiratory and hemodynamic variables

**Fig. 5:**
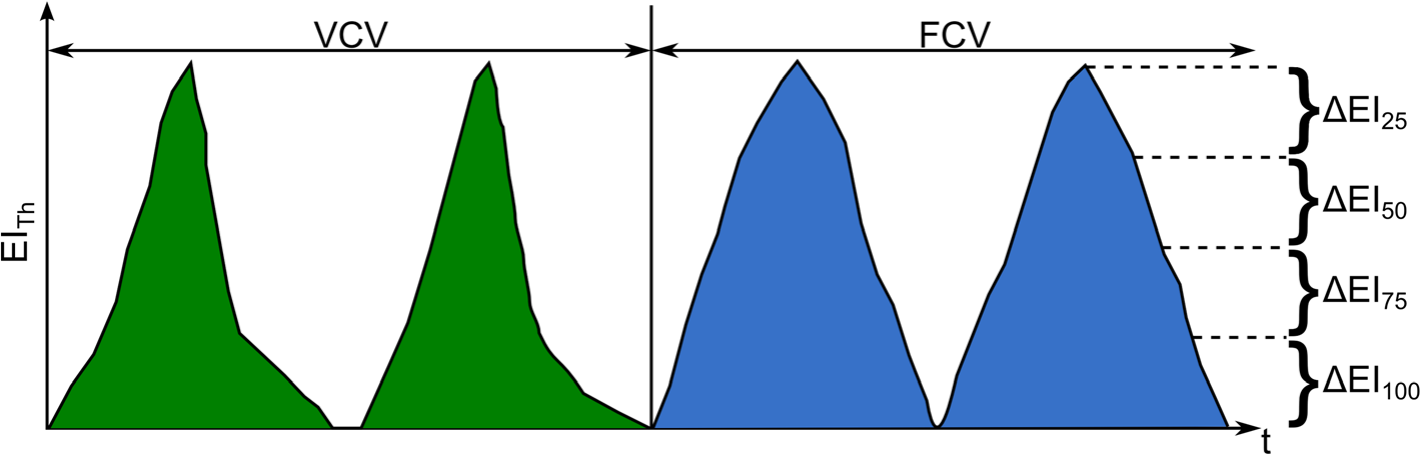
Exemplary global thoracic electrical impedance (EI_Th_) during two tidal breathes of flow-controlled ventilation (FCV) and volume-controlled ventilation (VCV) in one obese patient. For further comparison, decrease in impedance during expiration was separated into four equal sections (ΔEI_25_, ΔEI_50_, ΔEI_75_ and ΔEI_100_) and matched with simultaneous tidal changes (comp. Fig. 6).

**Fig. 6:**
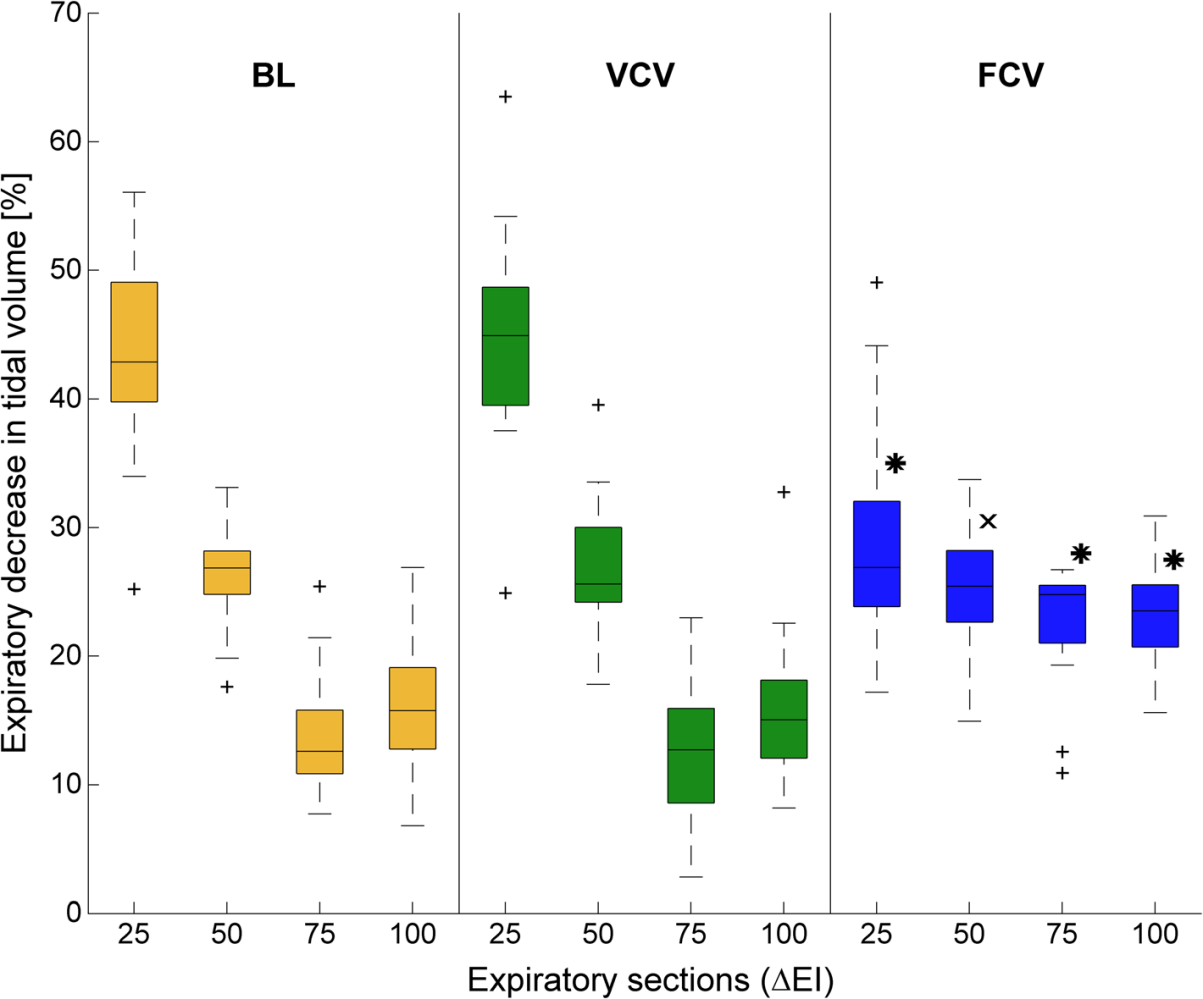
Relative expiratory decrease in tidal volume during the previously defined sections using the electrical impedance tomography (EIT) for volume-controlled baseline ventilation (BL), volume-controlled ventilation (VCV) and flow-controlled ventilation (FCV). In brief: the decline in global electrical thoracic impedance was separated into four equal sections (ΔEI_25_, ΔEI_50_, ΔEI_75_ and ΔEI_100_) (compare Fig. 5) and matched with the tidal changes simultaneously. On each box, the central mark indicates the second quartile, the bottom and top edges indicate quartiles (25th percentile and 75th percentile). On each box, the whiskers indicate the most extreme data points. Outliers are plotted individually (‘+’). * = *p* < 0.001 for baseline vs. FCV and VCV vs. FCV, ^x^ = *p* > 0.05 for baseline vs. FCV and VCV vs. FCV. Linear mixed effect model analyses were used to check for differences between the ventilation phases using R based software (jamovi project 2018, version 0.9.2.3). The randomization had no significant effect on the measured difference in end-expiratory lung volume between the ventilation phases

FCV was characterized by a more even decay of impedance throughout the expiration phase (Fig. 5). ΔEI_25_, ΔEI_50_, ΔEI_75_ and ΔEI_100_ showed a more even decrease during FCV compared to VCV (Fig. 6). ΔEI_25_ decreases about 45% during baseline ventilation and VCV and 25% during FCV. ΔEI_50_ showed no differences between the ventilation modes. ΔEI_75_ and ΔEI_100_ showed a lower decrease in global thoracic electrical impedance during baseline ventilation and VCV compared to FCV (Fig. 6).

## Discussion

In this study, we compared respiratory system mechanics and regional ventilation in obese patients during short application of FCV and VCV. The main findings of our study are that in obese patients, ΔEELV and mean lung volume decreased less during FCV than during VCV – even with identical respiratory and hemodynamic variables.

These effects were comparable to the effects one would expect from a PEEP increase and/or a tidal volume increase. However, minimal and maximal airway pressure and tidal volume remained unchanged. Our results are consistent with and enlarge upon earlier findings on the implications of a linearized expiratory pressure decrease in lung-healthy patients, lung healthy pigs and a porcine lung-injury model [6–9].

We observed the changes of respiratory mechanics during the VCV and FCV phases. Since baseline measurements were performed prior to the following ventilation sequence, we attribute the observed differences between baseline and VCV to the general tendency of the respiratory system to continued derecruitment during mechanical ventilation [18] which may be more pronounced in obese patients. The implications of obesity on respiratory system mechanics are well known: chest wall mechanics are impaired, and respiratory system compliance is reduced. Obese patients have an increased risk for early expiratory alveolar collapse and potential consecutive atelectrauma [3, 5, 17, 19, 20] and thus for decreased functional residual capacity and expiratory reserve volume [5, 13, 21, 22]. Therefore, besides low tidal volume and optional recruitment maneuvers, lung-protective ventilation strategies include the application of adequate PEEP in these patients. However, the ideal adjustment of applied tidal volume, and PEEP — with respect to the potential injurious effects of alveolar overdistension — in obese patients still remain obscure [20]. In this regard, FCV improved lung recruitment without modifying PEEP or tidal volume. Further, it should be noted that because of the controlled end-expiratory pressure during FCV, intrinsic PEEP is nearly excluded. Caused by the active control of the expiration phase, FCV is able to overcome the usually passively driven expiration during conventional ventilation.

The mechanisms behind this recruiting effects may be time dependent: when the lung volume falls below the closing capacity airway closure can occur within the expiration [5, 23]. In obese patients, this airway closure can be observed frequently [3]. The overall delayed expiration during FCV delays the time point at which the lung volume falls below the closing capacity. Consequently, the time until the lung volume exceeds closing capacity within the next inspiration is reduced and thus the risk of airway closure may be lowered [22]. The characterization and correlation between the expiratory decrease in global electrical impedance and expiratory decrease in intrapulmonary air and the increased mean airway pressure during expiration support this conjecture.

Theoretical and clinical observations predict that the linearized decrease in expiratory airway pressure has a beneficial impact on the intrapulmonary inhomogeneity [6, 7, 9, 17, 24]. However, the comparison of tidal impedance variation revealed no differences in intrapulmonary gas distribution during the different ventilation phases. The reduced accessibility of EIT images in obese and morbidly obese patients was described earlier and may be caused by the excessive volume of fat tissue around the chest wall. In horizontal supine position, this fat tissue moves laterally and may create potential shortcuts for the electrical currents of the EIT [25]. Therefore, the resolution of the EIT is limited, which may have masked differences in intrapulmonary inhomogeneity in our patients.

C_RS_ did not differ significantly between the investigated ventilation conditions. Reduced C_RS_ in obese patients may be caused mainly by excess adipose tissue around the chest wall and poor posture caused by thoracic kyphosis and lumbar hyperlordosis, aggravated through excessive abdominal fat tissue [19]. Under these conditions, the recruiting effect of FCV may have influenced C_RS_ only to a minor extent. This hypothesis is supported by earlier investigations that demonstrated that the intratidal recruitment state might differ without affecting C_RS_ [26, 27]_._ To investigate potential effects of FCV on the C_RS_ in obese patients, longer application of FCV may be necessary.

### Limitations of the study

We did not perform arterial blood gas analyses to examine the effects of FCV on gas exchange in our patients. In preclinical [17] and clinical studies [8], the controlled expiration improved oxygenation and CO_2_ elimination. However, in contrast to other centers, placing an arterial line is not part of our standard treatment in this patient group. Therefore, we felt that such invasive approach was not justified for our study. Further, it should be stated that duration of ventilation of 7 min in each ventilation mode is too short to fully evaluate the effects of FCV on regional ventilation. It follows that further studies are required to investigate the long-term effects of FCV on measurements of regional ventilation, respiratory and hemodynamic variables in obese patients.

## Conclusion

This is the first study to investigate the influence of FCV on respiratory mechanics and regional ventilation and in obese and morbidly obese patients. Utilizing measurement of regional ventilation, we could demonstrate that the linearized expiratory flow during FCV provided better maintenance of lung aeration with comparable tidal volume, P_Plat_ and PEEP, compared to VCV. The recruiting effect caused by the linearized expiratory air flow and the elevated P_mean_ during FCV may help prevent atelectasis and hypoxemia during mechanical ventilation in obese patients.

## Data Availability

The datasets used and analyzed during the current study are available from the corresponding author on request. Please note that EIT data files require large memory. A separate data transfer service will be used to transfer EIT data files.

## Abbreviations

ASA: American Society of Anesthesiologists
BMI: Body mass index
C_RS_: Quasi-static compliance of the respiratory system
EELV: End-expiratory lung volume
EIT: Electrical impedance tomography
FCV: Flow-controlled ventilation
MLV: Mean lung volume
PEEP: Positive end-expiratory pressure
P_mean_: Mean airway pressure
P_Plat_: Plateau pressure
SpO_2_: Peripheral oxygen saturation (pulse oximetry)
VCV: Volume-controlled ventilation
